# Dimeth­yl(2-oxo-2-phenyl­eth­yl)sulfanium bromide

**DOI:** 10.1107/S1600536810046404

**Published:** 2010-11-17

**Authors:** Zhiling Cao, Weiwei Liu, Fujun Yin

**Affiliations:** a School of Chemical Engineering, Huaihai Institute of Technology, Lianyungang 222005, People’s Republic of China; b Jiangsu Marine Resources Development Research Institute, Lianyungang 222005, People’s Republic of China

## Abstract

Single crystals of the title compound, C_10_H_13_OS^+^·Br^−^, were obtained from ethyl acetate/ethyl ether after reaction of acetophenone with hydro­bromic acid and dimethyl­sulfoxide. The carbonyl group is almost coplanar with the neighbouring phenyl ring [O—C—C—C = 178.9 (2)°]. The sulfanium group shows a trigonal–pyramidal geometry at the S atom. The crystal structure is stabil­ized by C—H⋯Br hydrogen-bonding inter­actions. Weak π–π inter­actions link adjacent phenyl rings [centroid–centroid distance = 3.946 (2) Å].

## Related literature

For applications of phenacyl sulfanium salts in organic synthesis, see: Crivello *et al.* (2000 ▶); Hirano *et al.* (2001 ▶). For related structures, see: Dossena *et al.* (1983 ▶); Svensson *et al.* (1996 ▶).
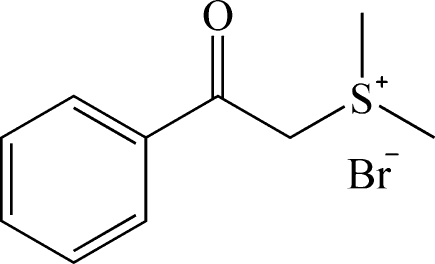


## Experimental

### 

#### Crystal data

C_10_H_13_OS^+^·Br^−^
*M* *_r_* = 261.17
Orthorhombic, 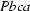

*a* = 15.7951 (17) Å
*b* = 7.4122 (8) Å
*c* = 19.007 (2) Å
*V* = 2225.3 (4) Å^3^
*Z* = 8
Mo *K*α radiation
μ = 3.84 mm^−1^
*T* = 296 K
0.40 × 0.38 × 0.25 mm

#### Data collection

Bruker APEXII CCD diffractometer
Absorption correction: multi-scan (*SADABS*; Bruker, 2005 ▶) *T*_min_ = 0.309, *T*_max_ = 0.447
16148 measured reflections
2294 independent reflections
1840 reflections with *I* > 2σ(*I*)
*R*_int_ = 0.034

#### Refinement

*R*[*F* ^2^ > 2σ(*F* ^2^)] = 0.027
*wR*(*F* ^2^) = 0.070
*S* = 1.04
2294 reflections
121 parameters
H-atom parameters constrained
Δρ_max_ = 0.47 e Å^−3^
Δρ_min_ = −0.40 e Å^−3^

### 

Data collection: *APEX2* (Bruker, 2005 ▶); cell refinement: *SAINT* (Bruker, 2005 ▶); data reduction: *SAINT*; program(s) used to solve structure: *SHELXS97* (Sheldrick, 2008 ▶); program(s) used to refine structure: *SHELXL97* (Sheldrick, 2008 ▶); molecular graphics: *ORTEP-3* (Farrugia, 1997 ▶) and *DIAMOND* (Brandenburg, 2010 ▶); software used to prepare material for publication: *publCIF* (Westrip, 2010 ▶).

## Supplementary Material

Crystal structure: contains datablocks I, global. DOI: 10.1107/S1600536810046404/fb2228sup1.cif

Structure factors: contains datablocks I. DOI: 10.1107/S1600536810046404/fb2228Isup2.hkl

Additional supplementary materials:  crystallographic information; 3D view; checkCIF report

## Figures and Tables

**Table 1:** Hydrogen-bond geometry (Å, °)

*D*—H⋯*A*	*D*—H	H⋯*A*	*D*⋯*A*	*D*—H⋯*A*
C4—H4⋯Br1^i^	0.93	2.92	3.844 (2)	171
C9—H9*C*⋯Br1^ii^	0.96	2.89	3.689 (2)	142

Symmetry codes: (i) 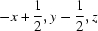; (ii) 
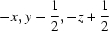
.

**Fig. 1.**
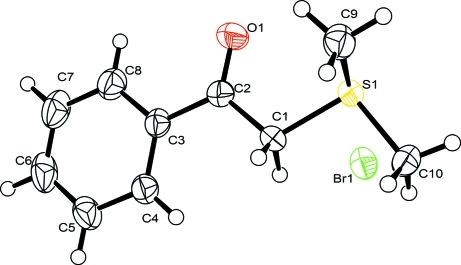
The molecular structure of the title compound with the atom-labeling scheme. Displacement ellipsoids are drawn at the 50% probability level.

**Fig. 2.**
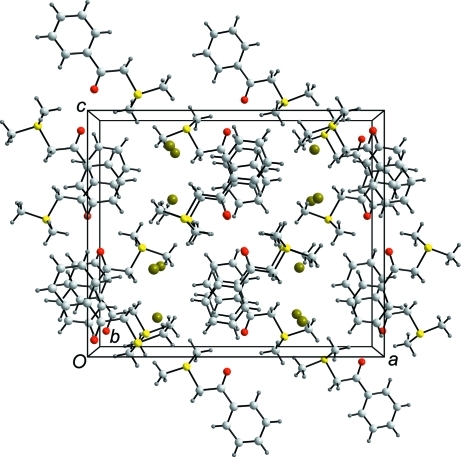
The crystal structure of the title compound, viewed along the b axis.

## supplementary crystallographic information

### Comment

sulphanium salts, characterized by a low sulphur valence and relatively unstable carbon-sulphur bonds, have found a broad practical application in organic chemistry. For example, dimethylphenacylsulphanium salts have been used for synthesis of a new class of photoinitiators for cationic polymerization (Crivello *et al.*, 2000) as well as of novel fluorophores (Hirano *et al.*, 2001). In the crystal structure of the title complex (Fig. 1), the phenyl ring is coplanar with the carbonyl group. The sulphanium group shows a trigonal-pyramidal geometry. All the bond lengths and bond angles are within the normal range (Dossena *et al.*, 1983; Svensson *et al.*, 1996).

There are C—H···Br hydrogen-bond interactions that stabilize the crystal structure (Tab. 1, Fig. 2). Weak π-electron ring - π-electron ring interactions between the phenyl rings that are stacked along the *b* axis [the centroid-centroid distance equals to 3.946 (2) Å] are also present in the structure. The symmetry codes for each of the adjacent rings: 1/2-*x*,-1/2+*y*,*z*; 1/2-*x*,1/2+*y*,*z*.

### Experimental

Acetophenone (0.05 mol) was dissolved in a mixture of 48% (w%) aqueous hydrobromic acid (20 ml) and dimethylsulfoxide (40 ml). This solution was heated under reflux for 5 h to afford the title compound. The mixture was extracted three times, each time with 25 ml of ethyl acetate. Ethyl ether (15 ml) was added to the combined organic extracts. The solution was allowed to stand overnight. After filtration and washing with ethyl ether, colourless needle-shaped crystals were obtained. The crystals were as long as 13 mm being thick of about 0.4 mm.

### Refinement

All the hydrogens could have been discerned in the difference electron map. However, the hydrogens were situated into the idealized postions and treated in the riding mode approximation. The used constraints were as follows: C—H = 0.93 (aryl C), C—H = 0.97 (methylene C), C—H = 0.96 Å (methyl C). *U*_iso_(H) = 1.2*U*_eq_(C_aryl_/C_methylene_), *U*_iso_(H) = 1.5*U*_eq_(C_methyl_).

### Crystal data

**Table:**

C_10_H_13_OS^+^·Br^−^	*D*_x_ = 1.559 Mg m^−^^3^
*M**_r_* = 261.17	Melting point: 531 K
Orthorhombic, *P**b**c**a*	Mo *K*α radiation, λ = 0.71073 Å
Hall symbol: -P 2ac 2ab	Cell parameters from 5457 reflections
*a* = 15.7951 (17) Å	θ = 2.5–26.9°
*b* = 7.4122 (8) Å	µ = 3.84 mm^−^^1^
*c* = 19.007 (2) Å	*T* = 296 K
*V* = 2225.3 (4) Å^3^	Plate, colourless
*Z* = 8	0.40 × 0.38 × 0.25 mm
*F*(000) = 1056	

### Data collection

**Table:**

Bruker APEXII CCD diffractometer	2294 independent reflections
Radiation source: fine-focus sealed tube	1840 reflections with *I* > 2σ(*I*)
graphite	*R*_int_ = 0.034
φ and ω scans	θ_max_ = 26.5°, θ_min_ = 2.1°
Absorption correction: multi-scan (*SADABS*; Bruker, 2005)	*h* = −18→19
*T*_min_ = 0.309, *T*_max_ = 0.447	*k* = −9→9
16148 measured reflections	*l* = −23→23

### Refinement

**Table:**

Refinement on *F*^2^	Secondary atom site location: difference Fourier map
Least-squares matrix: full	Hydrogen site location: difference Fourier map
*R*[*F*^2^ > 2σ(*F*^2^)] = 0.027	H-atom parameters constrained
*wR*(*F*^2^) = 0.070	*w* = 1/[σ^2^(*F*_o_^2^) + (0.0328*P*)^2^ + 1.2316*P*] where *P* = (*F*_o_^2^ + 2*F*_c_^2^)/3
*S* = 1.04	(Δ/σ)_max_ < 0.001
2294 reflections	Δρ_max_ = 0.47 e Å^−^^3^
121 parameters	Δρ_min_ = −0.39 e Å^−^^3^
0 restraints	Extinction correction: *SHELXL97* (Sheldrick, 2008), Fc^*^=kFc[1+0.001xFc^2^λ^3^/sin(2θ)]^-1/4^
50 constraints	Extinction coefficient: 0.0092 (5)
Primary atom site location: structure-invariant direct methods	

### Special details

**Table:**

Geometry. All esds (except the esd in the dihedral angle between two l.s. planes) are estimated using the full covariance matrix. The cell esds are taken into account individually in the estimation of esds in distances, angles and torsion angles; correlations between esds in cell parameters are only used when they are defined by crystal symmetry. An approximate (isotropic) treatment of cell esds is used for estimating esds involving l.s. planes.
Refinement. Refinement of *F*^2^ against ALL reflections. The weighted *R*-factor *wR* and goodness of fit *S* are based on *F*^2^, conventional *R*-factors *R* are based on *F*, with *F* set to zero for negative *F*^2^. The threshold expression of *F*^2^ > σ(*F*^2^) is used only for calculating *R*-factors(gt) *etc*. and is not relevant to the choice of reflections for refinement. *R*-factors based on *F*^2^ are statistically about twice as large as those based on *F*, and *R*- factors based on ALL data will be even larger.

### Fractional atomic coordinates and isotropic or equivalent isotropic displacement parameters (Å^2^)

**Table:**

	*x*	*y*	*z*	*U*_iso_*/*U*_eq_	
Br1	0.143976 (16)	0.50139 (3)	0.223269 (13)	0.04541 (12)	
O1	0.07571 (10)	0.1820 (3)	0.03073 (8)	0.0522 (5)	
S1	0.06002 (3)	0.08052 (8)	0.17220 (3)	0.03120 (15)	
C1	0.15917 (13)	0.0825 (3)	0.12611 (11)	0.0335 (5)	
H1A	0.1988	0.1608	0.1504	0.040*	
H1B	0.1828	−0.0383	0.1254	0.040*	
C2	0.14674 (13)	0.1486 (3)	0.05159 (11)	0.0338 (5)	
C3	0.22286 (13)	0.1698 (3)	0.00714 (11)	0.0331 (5)	
C4	0.30359 (14)	0.1336 (3)	0.03261 (12)	0.0392 (5)	
H4	0.3108	0.0926	0.0785	0.047*	
C5	0.37317 (15)	0.1589 (4)	−0.01063 (14)	0.0507 (7)	
H5	0.4273	0.1349	0.0062	0.061*	
C6	0.36212 (17)	0.2195 (4)	−0.07849 (15)	0.0568 (8)	
H6	0.4091	0.2377	−0.1071	0.068*	
C7	0.28266 (18)	0.2535 (4)	−0.10436 (14)	0.0553 (7)	
H7	0.2758	0.2924	−0.1505	0.066*	
C8	0.21302 (16)	0.2300 (3)	−0.06179 (12)	0.0447 (6)	
H8	0.1592	0.2543	−0.0791	0.054*	
C9	0.01019 (16)	−0.1180 (3)	0.13854 (12)	0.0437 (6)	
H9A	0.0471	−0.2196	0.1449	0.066*	
H9B	−0.0014	−0.1024	0.0893	0.066*	
H9C	−0.0419	−0.1388	0.1633	0.066*	
C10	0.09574 (16)	0.0056 (3)	0.25659 (12)	0.0411 (6)	
H10A	0.1271	−0.1045	0.2513	0.062*	
H10B	0.0478	−0.0151	0.2866	0.062*	
H10C	0.1314	0.0962	0.2772	0.062*	

### Atomic displacement parameters (Å^2^)

**Table:**

	*U*^11^	*U*^22^	*U*^33^	*U*^12^	*U*^13^	*U*^23^
Br1	0.04414 (17)	0.04672 (18)	0.04538 (17)	−0.00352 (11)	0.00865 (10)	−0.00266 (11)
O1	0.0314 (9)	0.0831 (13)	0.0423 (9)	0.0042 (9)	−0.0031 (7)	0.0146 (9)
S1	0.0270 (3)	0.0370 (3)	0.0295 (3)	0.0031 (2)	0.0022 (2)	−0.0003 (2)
C1	0.0234 (10)	0.0458 (13)	0.0313 (11)	0.0017 (10)	0.0013 (8)	−0.0004 (10)
C2	0.0309 (12)	0.0390 (12)	0.0315 (11)	−0.0021 (9)	−0.0010 (9)	−0.0005 (10)
C3	0.0344 (11)	0.0347 (12)	0.0301 (10)	−0.0071 (10)	0.0032 (9)	−0.0041 (9)
C4	0.0342 (12)	0.0477 (14)	0.0358 (12)	−0.0047 (10)	0.0036 (9)	−0.0060 (10)
C5	0.0334 (13)	0.0658 (18)	0.0528 (15)	−0.0105 (12)	0.0081 (11)	−0.0180 (13)
C6	0.0515 (16)	0.0684 (19)	0.0506 (15)	−0.0242 (14)	0.0231 (12)	−0.0159 (14)
C7	0.0677 (18)	0.0636 (18)	0.0345 (12)	−0.0147 (15)	0.0112 (12)	0.0022 (12)
C8	0.0470 (14)	0.0511 (15)	0.0360 (12)	−0.0070 (12)	0.0014 (11)	0.0009 (11)
C9	0.0435 (13)	0.0475 (15)	0.0402 (13)	−0.0119 (11)	0.0050 (10)	−0.0053 (10)
C10	0.0441 (14)	0.0513 (15)	0.0278 (11)	0.0054 (11)	−0.0001 (10)	0.0027 (10)

### Geometric parameters (Å, °)

**Table:**

O1—C2	1.215 (3)	C5—H5	0.9300
S1—C9	1.788 (2)	C6—C7	1.371 (4)
S1—C10	1.789 (2)	C6—H6	0.9300
S1—C1	1.794 (2)	C7—C8	1.377 (4)
C1—C2	1.511 (3)	C7—H7	0.9300
C1—H1A	0.9700	C8—H8	0.9300
C1—H1B	0.9700	C9—H9A	0.9600
C2—C3	1.478 (3)	C9—H9B	0.9600
C3—C4	1.390 (3)	C9—H9C	0.9600
C3—C8	1.393 (3)	C10—H10A	0.9600
C4—C5	1.385 (3)	C10—H10B	0.9600
C4—H4	0.9300	C10—H10C	0.9600
C5—C6	1.377 (4)		
			
C9—S1—C10	101.78 (12)	C7—C6—C5	120.8 (2)
C9—S1—C1	102.49 (11)	C7—C6—H6	119.6
C10—S1—C1	99.50 (11)	C5—C6—H6	119.6
C2—C1—S1	110.29 (15)	C6—C7—C8	119.8 (2)
C2—C1—H1A	109.6	C6—C7—H7	120.1
S1—C1—H1A	109.6	C8—C7—H7	120.1
C2—C1—H1B	109.6	C7—C8—C3	120.3 (2)
S1—C1—H1B	109.6	C7—C8—H8	119.9
H1A—C1—H1B	108.1	C3—C8—H8	119.9
O1—C2—C3	122.9 (2)	S1—C9—H9A	109.5
O1—C2—C1	119.45 (19)	S1—C9—H9B	109.5
C3—C2—C1	117.68 (18)	H9A—C9—H9B	109.5
C4—C3—C8	119.5 (2)	S1—C9—H9C	109.5
C4—C3—C2	121.77 (19)	H9A—C9—H9C	109.5
C8—C3—C2	118.8 (2)	H9B—C9—H9C	109.5
C5—C4—C3	119.7 (2)	S1—C10—H10A	109.5
C5—C4—H4	120.2	S1—C10—H10B	109.5
C3—C4—H4	120.2	H10A—C10—H10B	109.5
C6—C5—C4	120.0 (2)	S1—C10—H10C	109.5
C6—C5—H5	120.0	H10A—C10—H10C	109.5
C4—C5—H5	120.0	H10B—C10—H10C	109.5
			
C9—S1—C1—C2	77.33 (19)	C8—C3—C4—C5	0.4 (3)
C10—S1—C1—C2	−178.25 (17)	C2—C3—C4—C5	−178.7 (2)
S1—C1—C2—O1	−3.6 (3)	C3—C4—C5—C6	0.0 (4)
S1—C1—C2—C3	176.26 (17)	C4—C5—C6—C7	−0.8 (4)
O1—C2—C3—C4	178.9 (2)	C5—C6—C7—C8	1.2 (4)
C1—C2—C3—C4	−1.0 (3)	C6—C7—C8—C3	−0.7 (4)
O1—C2—C3—C8	−0.3 (4)	C4—C3—C8—C7	−0.1 (4)
C1—C2—C3—C8	179.8 (2)	C2—C3—C8—C7	179.2 (2)

### Hydrogen-bond geometry (Å, °)

**Table:**

*D*—H···*A*	*D*—H	H···*A*	*D*···*A*	*D*—H···*A*
C4—H4···Br1^i^	0.93	2.92	3.844 (2)	171
C9—H9C···Br1^ii^	0.96	2.89	3.689 (2)	142

Symmetry codes: (i) −*x*+1/2, *y*−1/2, *z*; (ii) −*x*, *y*−1/2, −*z*+1/2.
